# Evaluation of zygomaticus major muscle and lip-closing force in orthognathic surgery: retrospective study

**DOI:** 10.1186/s40902-025-00466-9

**Published:** 2025-05-21

**Authors:** Riku Kohara, Karen Gomi, Young-Min Shin, Akinori Moroi, Kunio Yoshizawa, Koichiro Ueki

**Affiliations:** 1 Department of Oral and Maxillofacial Surgery, Division of Medicine, Interdisciplinary Graduate School, University of Yamanashi, Yamanashi, Japan; 2 Director of Dept. of Dentistry (Oral & Maxillofacial surgery), Dongsan medical center, Keimyung university, School of medicine, Dalgubeoldaero 1035, Dalseogu, Daegu Korea

**Keywords:** Orthognathic surgery, Zygomaticus major muscle, Lip-closing force, CT value

## Abstract

**Background:**

This study evaluated the zygomaticus major muscle and lip-closing force before and after orthognathic surgery. Sixty female patients with jaw deformities who underwent orthognathic surgery were included. Lip-closing force and computed tomography (CT) assessments were conducted preoperatively and 1 year postoperatively. Lip pressure was measured using the Lip De Cum LDC-110R® (Cosmos Instruments Co., Ltd., Tokyo, Japan). CT images were reconstructed using ProPlan CMF (Materialize, Belgium), and zygomaticus major muscle width and CT values were analyzed. To the best of our knowledge, no previous studies have employed CT values to assess muscles in the oral and maxillofacial area.

**Results:**

In both class II and class III females, postoperative zygomaticus major muscle width was significantly higher than preoperative values. In class II females, postoperative zygomaticus major muscle CT values were also significantly higher than preoperative values. Simple linear regression analysis with age as the dependent variable revealed significant associations between pre- and postoperative zygomaticus major muscle widths in both groups. Additionally, simple linear regression analysis with CT values as the dependent variable demonstrated significant associations with postoperative lip-closing force in both class II and class III females.

**Conclusions:**

This study suggests that orthognathic surgery significantly modifies the zygomaticus major muscle morphology and function, impacting CT values.

## Background

The lip-closing function involves various movements that differ in type and intensity, depending on physiological needs. These movements are more complex and coordinated than simple actions such as blinking, engaging in orbicularis oris and other facial muscles, which have muscle fibers arranged in multiple directions around the mouth [2].

The zygomaticus major originates from the posterior part of the lateral surface of the zygomatic bone and is inserted at the corner of the mouth. It is a facial expression muscle responsible for elevating the corners of the mouth upward and outward [6]. Along with the orbicularis and buccal muscles, it forms a modiolus at the angle of the mouth and acts as an antagonist to lip-closing forces. Studies have reported that the zygomatic major muscle undergoes a reduction in length and an increase in width and thickness during the expression of joy [4]. Muscle thickness is considered the most reliable parameter for accurately evaluating facial muscle function. Therefore, evaluating the zygomaticus major muscle may be useful for preoperative and postoperative assessments in orthognathic surgery and other scenarios requiring accurate functional assessment of facial expression muscles.

Computed tomography (CT) and magnetic resonance imaging (MRI) are effective tools for assessing muscle function. CT scans provide objective measurements of skeletal muscle mass using CT values. However, no previous studies have employed CT values to assess muscles in the oral and maxillofacial area .

Therefore, this study aimed to evaluate the zygomaticus major muscle and lip-closing force following orthognathic surgery.

## Methods

### Study design and participants

This retrospective study included 60 female patients who underwent Le Fort I osteotomy (L-I) or sagittal split ramus osteotomy (SSRO) for jaw deformities at the University of Yamanashi Hospital between January 2019 and December 2024. Patients with craniofacial trauma, congenital anomalies, occlusal interference due to prosthesis, or signs of temporomandibular joint disorder were excluded. This study was approved by the Clinical Research Ethics Committee of the University of Yamanashi Hospital (approval number: 2921) and adhered to the principles of the Declaration of Helsinki. Informed consent was obtained from all patients.

### Lip-closing force

Lip pressure was measured using the Lip De Cum LDC-110R® (Cosmos Instruments Co., Ltd., Tokyo, Japan), consisting of a sensor with a lip adapter and a digital display. Measurements were performed preoperatively (within one month before surgery) and 1 year postoperatively. Briefly, a lip holder (Ducklings®) was attached to the sensor, and the lip-closure strength indicator (Lip De Cum®^)^ was calibrated. Participants, seated upright with the Frankfort horizontal (FH) plane parallel to the floor, were instructed to close their upper and lower lips with maximum force to measure lip-closure strength. They were also asked to maintain upper and lower teeth contact [10]. During the 30-s measurement, waveforms were displayed on a personal computer (PC) connected to the Lip De Cum®, and the highest recorded value was considered the maximum lip-closing force [12–14]. Measurements were taken three times, and the mean value of each set of measurements was used. A 40-s rest period was allowed between measurements.

### Computed tomography (CT) measurements

CT imaging was conducted with patients positioned on a gantry parallel to the floor, ensuring that the FH plane was perpendicular. Participants were instructed to breathe normally and refrain from swallowing during the scan. A skilled radiologist performed the scans using a high-speed Advantage CT generator (LightSpeed Plus; GE Healthcare, Milwaukee, WI, USA), with sequences captured at 1.25-mm intervals (120 kV; average, 150 mA; 0.7 s/rotation; pitch factor: 0.75). The DICOM data were imported into ProPlan CMF (Materialize, Belgium) to construct three-dimensional models.

**Fig. 1:**
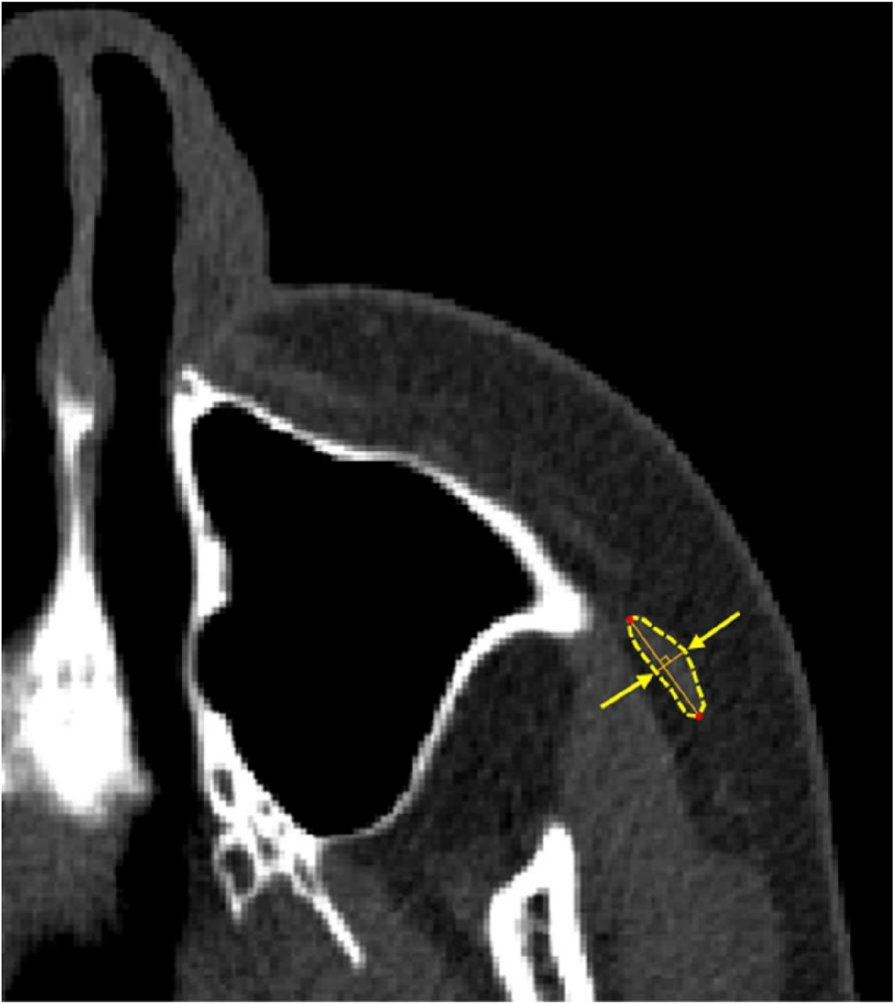
CT measurement for the zygomaticus major muscle. (1) Zygomaticus major muscle width. (2) CT value of the zygomaticus major muscle. CT, computed tomography

CT measurements followed a previously reported method [11]. The RL line was defined as the line between the most anterior points of the bilateral auricles in a plane parallel to the FH plane. Multiplanar reconstruction was used to establish an arbitrary plane parallel to the plane in which the RL line was determined. The horizontal plane above the mandibular foramen parallel to the FH plane was identified bilaterally, and the zygomaticus major muscle was measured on each side preoperatively and 1 year postoperatively, with the higher values recorded as the final measurements. CT values were measured using a circular region of interest (ROI) placed centrally within the muscle tissue, ensuring that partial volume effects did not influence the results. The average CT value of each pixel within the ROI was then calculated. Reference points were established as follows (Fig. 1):

1. Zygomaticus major muscle width: The maximum thickness of the zygomaticus major muscle measured parallel to the RL line
2. CT value of the zygomaticus major muscle: The mean CT value in the plane where the zygomaticus major muscle reaches its greatest diameter.

### Cephalograms mesurement

Lateral cephalograms were taken for all patients before and at 1 year after surgery for the skeletal analysis. Cephalograms were obtained using a standardized cephalometric technique and were analyzed using CephaloMetrics AtoZ software (Yasunaga Computer Systems Inc., Fukui, Japan) on a Microsoft computer.

### Statistical analysis

A correlation between lip-closing force and postoperative recovery of perioral muscles was anticipated in patients with jaw deformities. Using Fisher’s exact method with a 35% relative effect size, a two-sided alpha of 0.05, and 80% power, it was estimated that 30 individuals per group were needed.

Measurement repeatability was assessed using the intraclass correlation coefficient (ICC), which reflects the proportion of total measurement variance due to intersubject variability. CT parameters were measured at 10-day intervals, and ICCs were computed. The Shapiro–Wilk test was used to assess data normality. Preoperative and 1-year postoperative data were compared using paired sample *t*-tests. Linear regression analysis evaluated relationships between age, zygomaticus major muscle parameters, and lip-closing force. Statistical analyses were performed using IBM SPSS Statistics (version 26.0; IBM Corp., Armonk, NY, USA).

## Results

### Patient characteristics

Sixty patients aged 17–45 years (mean age: 24.7 years; standard deviation [SD]: 6.0) were included in this study. They were categorized into two groups: class II female (*n* = 30) and class III female (*n* = 30).

### Pre- and postoperative comparisons

**Fig. 2:**
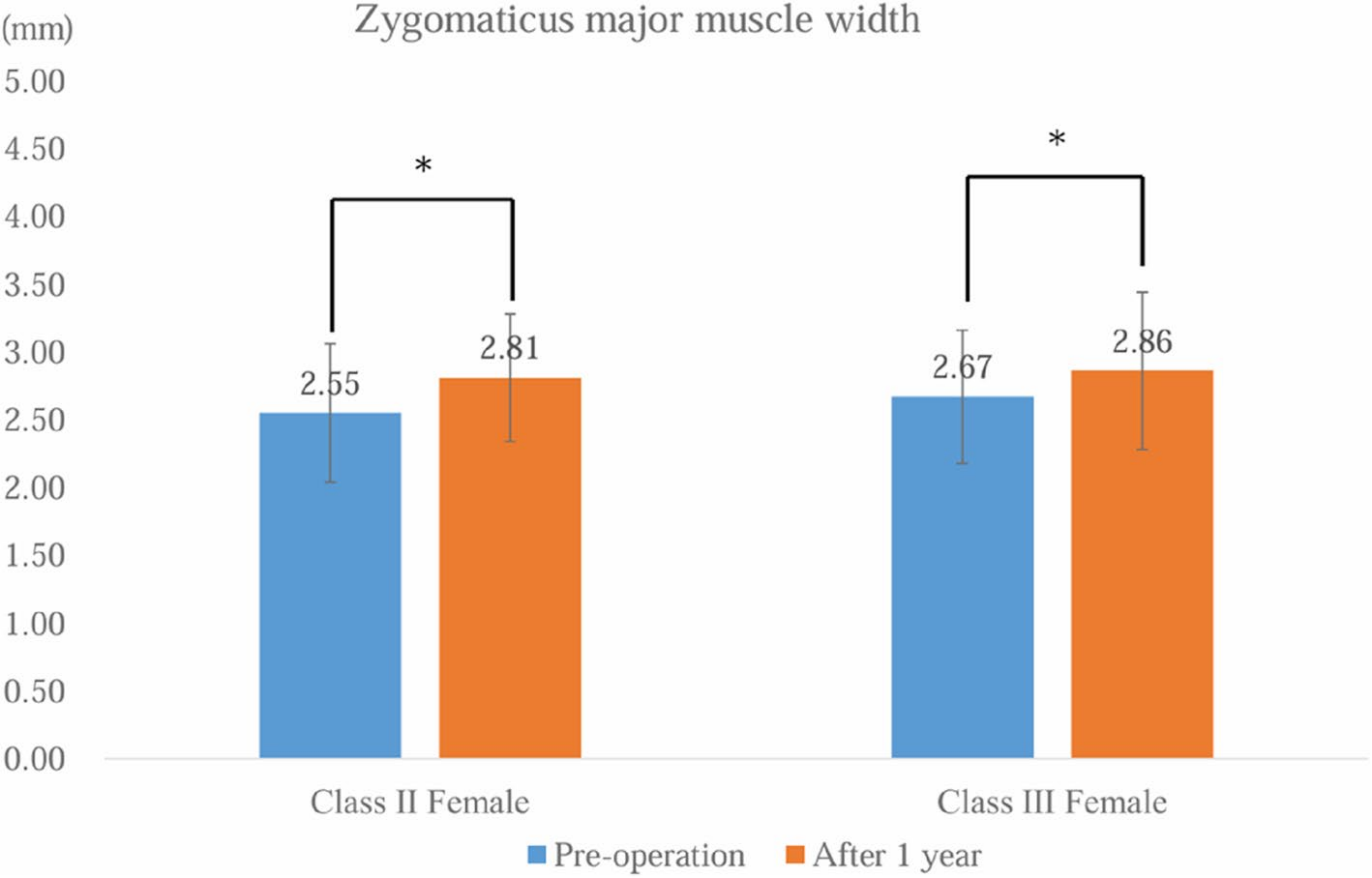
Zygomaticus major muscle width measurements. Error bars indicate standard deviation. *Significant difference at *p* < 0.05

**Fig. 3:**
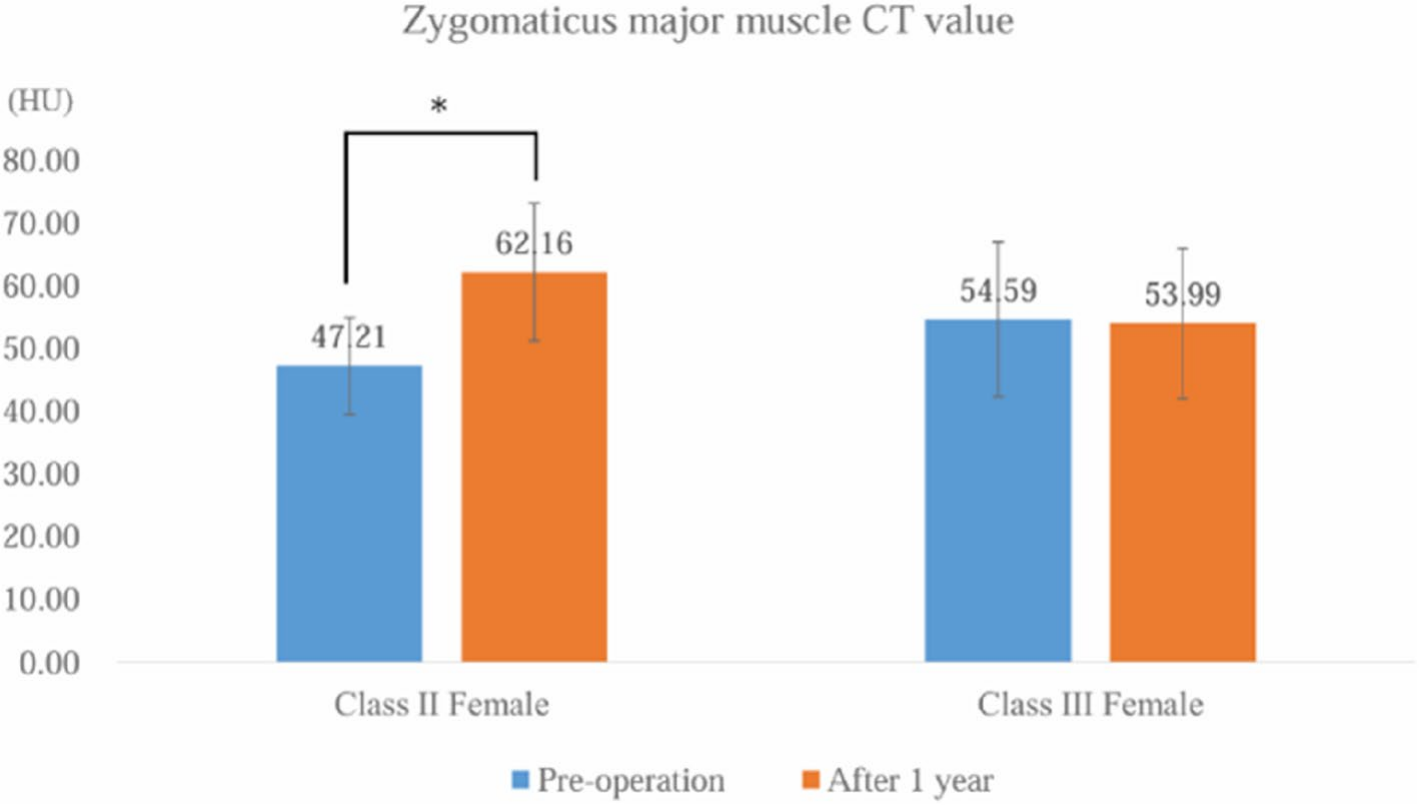
Zygomaticus major muscle CT value measurements. Error bars indicate standard deviation. *Significant difference at *p* < 0.05

Postoperative zygomaticus major muscle width was significantly greater than preoperative values in class II (*p* = 0.044) and class III females (*p* = 0.0020) (Fig. 2). In class II females, postoperative zygomaticus major muscle CT values were significantly higher than preoperative values (*p* < 0.001) (Fig. 3). In class II females, SNA (*p* < 0.001) and SNB (*p* < 0.001) were significantly smaller than postoperatively, while FMA (*p* = 0.002), ramus inclination (FH) (*p* < 0.001), and occlusal plane (*p* < 0.001) were significantly larger than postoperatively. In class III females, SNB (*p* = 0.005) and F. Ht. (ANS-Me) (*p* < 0.001) were significantly smaller than postoperatively, while gonial angle (*p* = 0.004) was significantly larger than postoperatively (Table 1).

**Table 1:** Result of cephalometric analysis

			SNA	SNB	FMA	Gonial angle	Ramus inclination (FH)	Occlusal plane	F. Ht (ANS-Me)
Class II female	Pre -operation	Mean	79*	72.01*	42.67*	128.00	94.67*	19.02*	66.58
		SD	5.06	4.61	7.84	7.59	6.38	5.07	7.37
	Post -operation	Mean	81.65*	75.70*	38.91*	130.36	88.55*	15.76*	67.17
		SD	4.65	4.22	6.41	7.56	6.96	4.18	6.82
Class III female	Pre -operation	Mean	81.16	81.32*	33.74	128.77*	84.97	13.68	72.54*
		SD	4.07	4.00	6.54	8.24	7.79	5.05	6.44
	Post -operation	Mean	82.22	79.15*	36.01	130.91*	85.10	12.54	68.75*
		SD	4.87	4.29	6.66	7.78	6.45	5.10	6.51

^*^p < 0.05

### Simple linear regression analysis with age as the dependent variable

Simple linear regression analysis, with age as the dependent variable, demonstrated significant associations between the preoperative and postoperative zygomaticus major muscle widths in both class II and III females.

**Fig. 4:**
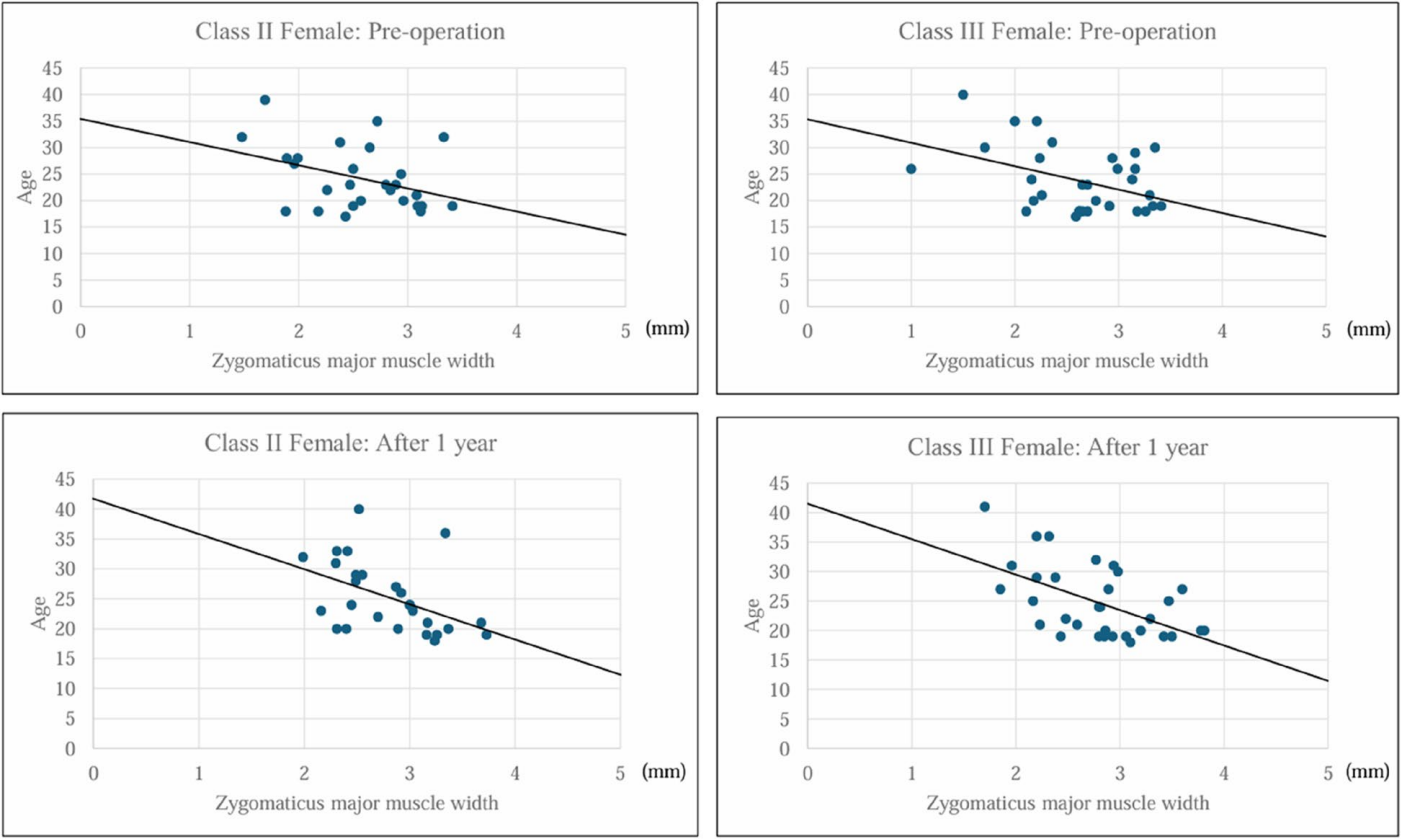
Simple linear regression analysis with age as the dependent variable

In class II females, the preoperative regression equation for age based on zygomaticus major muscle width was calculated as follows: age = (− 7.656 × pre-zygomaticus major muscle width) + 44.069 (*R*^2^ = 0.4761, *p* < 0.001). Postoperatively, the equation changed to the following: age = (− 5.875 × post-zygomaticus major muscle width) + 40.705 (*R*^2^ = 0.2125, *p* < 0.001), indicating a shift in the relationship following surgery. Similarly, in class III females, the preoperative regression equation was as follows: age = (− 5.447 × pre-zygomaticus major muscle width) + 38.175 (*R*^2^ = 0.2016, *p* = 0.011), whereas the postoperative equation was as follows: age = (− 6.012 × post-zygomaticus major muscle width) + 40.508 (*R*^2^ = 0.3025, *p* < 0.001). These findings suggest that orthognathic surgery influences the association between age and zygomaticus major muscle width, with notable postoperative adjustments in both class II and class III female patients (Fig. 4).

### Simple linear regression analysis with zygomatics major muscle CT value as the dependent variable

**Fig. 5:**
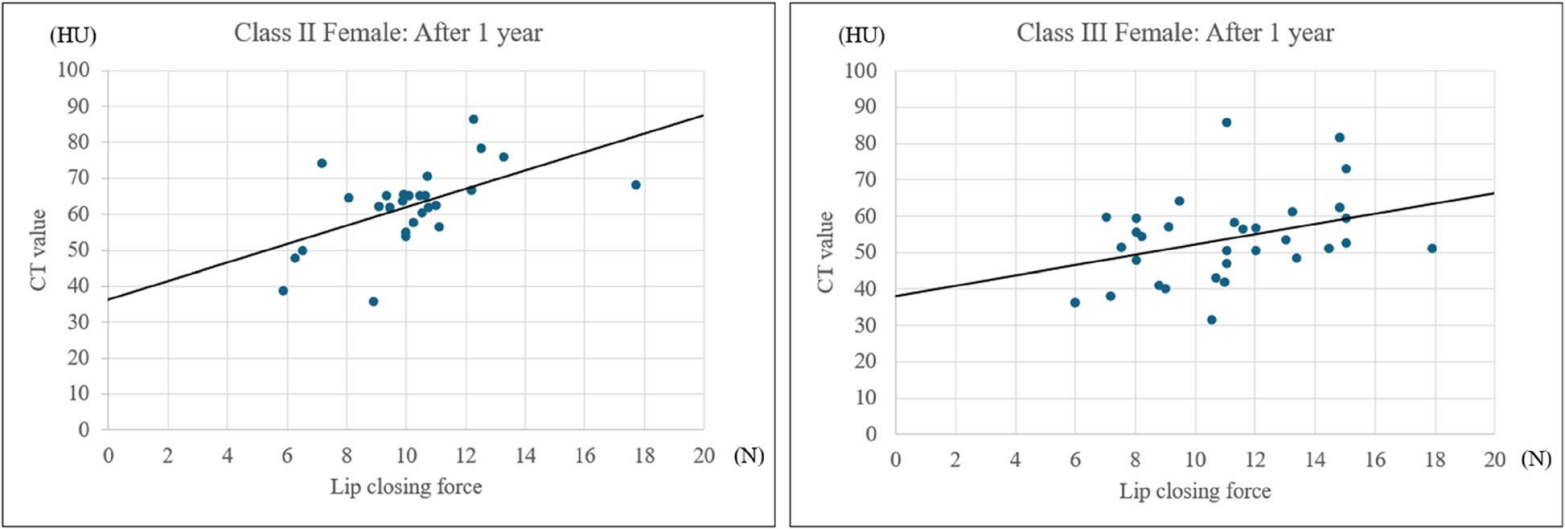
Simple linear regression analysis with zygomaticus major muscle CT value as the dependent variable

Simple linear regression analysis, with zygomatic major muscle CT value as the dependent variable, revealed significant correlations with postoperative lip-closing force in both class II and class III females. In class II females, the postoperative regression equation was as follows: postoperative CT value = (2.566 × post lip-closing force) + 36.191 (*R*^2^ = 0.3124, *p* = 0.002). Similarly, in class III females, the equation was as follows: postoperative CT value = (1.416 × post lip-closing force) + 38.100 (*R*^2^ = 0.1253, *p* = 0.047). These results indicate that improvements in lip-closing force following surgery correspond with changes in zygomaticus major muscle density, as reflected in CT values. This further supports the hypothesis that orthognathic surgery enhances perioral muscle function, contributing to improved postoperative outcomes (Fig. 5).

## Discussion

The significance of jaw deformity extends beyond aesthetic concerns, as it also plays a crucial role in functional improvement and psychological and social well-being. Orthognathic surgery not only enhances jaw appearance but also facilitates eating and speaking, improves jaw health, and enhances overall quality of life, making it an essential treatment for affected patients.

Several studies have investigated changes in lip-closure force following orthognathic surgery [5, 12–14]). Research has shown that the maximum lip-closing force at 6 months and 1 year postoperatively exceeds preoperative values in both sexes [10]. Additionally, studies on masticatory muscle changes before and after orthognathic surgery indicate significant improvements. For instance, in patients with class III dentofacial deformity, masseter muscle thickness increased 3 years after orthognathic surgery, approaching levels seen in control subjects [9]. In class II cases, masseter muscle length significantly increased 1 year postoperatively [7]

To the best of our knowledge, no previous studies have examined changes in the zygomaticus major muscle or its relationship with lip-closing force following orthognathic surgery. Lip-closing force primarily results from the coordinated action of the orbicularis oris and buccinator muscles; however, previous reports have also observed simultaneous contraction of the zygomaticus major muscles, which acts as an antagonist [8]. In our study, orthognathic surgery was associated with an increase in the maximum width of the zygomaticus major muscle in both class II and III females. Additionally, 1 year postoperatively, the mean CT value within the measurement range increased in class II females. Research has indicated that lip-closing force in class II cases is significantly lower than that in control groups [10], likely due to variations in the vertical skeletal pattern and upper incisor angulation. Our findings suggest that orthognathic surgery, including corrective procedures, improves skeletal structure and oral function in class II females, leading to enhanced labial closure force and improved function of the zygomaticus major muscle, which acts as an antagonist.

Attenuation of CT values has been previously reported and is influenced by three key components: lipids, glycogen, and water. Fat infiltration is widely recognized as the primary cause of reduced muscle attenuation, with lipid accumulation directly associated with lower muscle attenuation. Muscle lipids consist of various lipid species, including free fatty acids, diacylglycerols, triacylglycerols, and phospholipids. It is not only the quantity but also the composition of these lipid components that significantly impact muscle function. Recent studies suggest that lipid composition within muscles may be as crucial as total fat content in determining muscle performance [1]. Given that the muscle composition varies across different muscles, these differences should be reflected in CT values, which may also change with age and sex.

In the present study, we examined the CT values of facial muscles following orthognathic surgery. One year postoperatively, a positive correlation was observed between the mean CT values of the zygomaticus major muscle and lip-closing force in both class II and class III females. Consistent with previous studies, these findings suggest postoperative improvements in lip-closure strength occur in patients with jaw deformities and are associated with enhanced function of the zygomaticus major muscle, which acts as an antagonist.

The zygomaticus major plays a crucial role in facial expressions by elevating the corners of the mouth. In this study, a negative correlation was observed between the width of the zygomaticus major muscle and age, both preoperatively and 1 year postoperatively. This suggests that the width of the zygomaticus major muscle decreases with age. These results align with previous studies using surface-derived electromyography (EMG) measurements [3]. However, our study also found that in both class II and class III females, postoperative zygomaticus major muscle width increased, suggesting that corrective surgery enhances muscle function. This improvement may be an important factor for postoperative rehabilitation.

Cephalometric analysis revealed skeletal changes before and after surgery. These changes indicate that in both groups, the postoperative skeletal structure approached a normal configuration, which may help facilitate smooth contraction of the zygomaticus major muscle.

A limitation of this study is that all participants in the class II group were female, and lip-closing force data for male subjects in this class were not available. Consequently, it was not possible to compare sex differences in lip-closing force among class II patients. Despite this limitation, these results suggest that changes in the zygomaticus major muscle related to lip-closing force are associated with improved skeletal stability and occlusion following orthognathic surgery. However, further studies with larger sample sizes are necessary to validate these findings.

## Conclusion

To the best of our knowledge, this is the first study to employ CT values to assess muscles in the temporomandibular region. Our findings suggest that orthognathic surgery alters the morphology of the zygomaticus major muscle, leading to changes in CT values. In addition, postoperative modifications in the zygomaticus major muscle were associated with improvements in lip-closing force, further supporting the functional benefits of surgery.

## Data Availability

No datasets were generated or analysed during the current study.

## Abbreviations

CT: Computed tomography
MRI: Magnetic resonance imaging
L-I: Le Fort I osteotomy
SSRO: Sagittal split ramus osteotomy
FH: Frankfort horizontal
PC: Personal computer
ROI: Region of interest
ICC: Intraclass correlation coefficient
